# Estimating unrecorded human-caused mortalities of grizzly bears in the Flathead Valley, British Columbia, Canada

**DOI:** 10.7717/peerj.5781

**Published:** 2018-10-11

**Authors:** Bruce N. McLellan, Garth Mowat, Clayton T. Lamb

**Affiliations:** 1 BC Ministry of Forests, Lands and Natural Resource Operations and Rural Development, D’Arcy, BC, Canada; 2 Forest Sciences, BC Ministry of Forests, Lands and Natural Resource Operations and Rural Development, Nelson, BC, Canada; 3 Department of Earth and Environmental Sciences, University of BC-Okanagan, Kelowna, BC, Canada; 4 Department of Biological Sciences, University of Alberta, Edmonton, AB, Canada

**Keywords:** British Columbia, Grizzly bear, Human-caused mortality, *Ursus arctos*, Reporting rates, Cryptic killing, Unknown fates

## Abstract

Managing the number of grizzly bear (*Ursus arctos*) mortalities to a sustainable level is fundamental to bear conservation. All known grizzly bear deaths are recorded by management agencies but the number of human-caused grizzly bear deaths that are not recorded is generally unknown, causing considerable uncertainty in the total number of mortalities. Here, we compare the number of bears killed legally by hunters to the number killed by people for all other reasons, for bears wearing functioning radiocollars and for uncollared bears recorded in the British Columbia (BC) government mortality database for the Flathead Valley in southeast BC. Between 1980 and 2016, permitted hunters killed 10 collared bears and 12 (9 known, 3 suspected) were killed by people for other reasons. This ratio differed (*p* < 0.0001) from the uncollared bears in the government database where 71 were killed by hunters while only 10 were killed for other reasons. We estimate that 88% (95% CI; 67–96%) of the human-caused mortalities that were not by permitted hunters were unreported. The study area may have low reporting rates because it is >40 km on a gravel road from a Conservation Officer office, so reporting is difficult and there are no human residences so there is little concern of a neighbor contacting an officer. Our results are likely indicative of other places that are road-accessed but far from settlements. We discuss the implications of sampling individuals for collaring and the possible implications of wearing a collar on the animal’s fate.

## Introduction

Knowing how and why large mammals die is fundamental for effective management, conservation, and even developing land-use policies. Documenting cause of death, however, is often difficult. Some causes, such as legal hunting or the removal of conflict animals by agency personnel can easily and accurately be quantified, while other causes, such as natural deaths or vehicle/train collisions, may rarely be known without a sample of radiocollared individuals. Documenting illegal killing can be much more difficult even with radiocollared animals. Some people that illegally kill animals destroy radiocollars so animals being monitored seem to just disappear (McLellan et al., 1999; Goodrich et al., 2008). For radiocollared animals, these events have recently been called cryptic killing (Liberg et al., 2012). Although difficult to quantify, various forms of unreported killing may be the dominant cause of death of animals in some areas (McLellan et al., 1999; Chapron et al., 2008; Liberg et al., 2012; Sönnichsen et al., 2017; Treves et al., 2017).

Cause of death varies greatly among species and even among areas for each species. Of large mammals, bears (*Ursus* spp.) have unique characteristics that make unreported human-caused mortality common. Unlike most other large mammals, bears are readily attracted to human residences and temporary camps by a variety of food sources where they are sometimes shot and their deaths are not always reported (McLellan et al., 1999). Grizzly bears (*Ursus arctos*), particularly mothers with cubs, can be threatening to people so are sometimes shot in perceived self-defense and such cases may also not be reported (McLellan et al., 1999; Garshelis, Gibeau & Herrero, 2005; Mace et al., 2012). Furthermore, bears are poached for body parts in some areas (Mills & Servheen, 1994).

Although there is considerable uncertainty over their eventual fates, an effort to limit human-caused mortality within sustainable limits has been the cornerstone of grizzly bear conservation and management across much of their distribution (McLellan, 1998; Servheen, 1998; Garshelis, Gibeau & Herrero, 2005; Schwartz, Haroldson & White, 2010). All agencies in North America that manage grizzly bears maintain a database of bear mortalities dating back many decades. These data are used to direct management actions to most effectively reduce human-caused mortalities, keep the number of deaths below levels that may pose a conservation risk, as well as to estimate the number of bears that could be sustainably killed by hunters (McLellan et al., 2017). The number of unreported human-caused mortalities is unknown but evidence suggests that many deaths are not reported (McLellan et al., 1999; Cherry et al., 2002; Ciarniello et al., 2009; Lamb et al., 2017).

Here, we estimate the proportion and number of human-caused grizzly bear deaths in the British Columbia (BC) Flathead Valley that were not in the government database by comparing the cause of death of a radiocollared sample of bears to the cause of death of bears that were not radiocollared in the government database. In BC, both radiocollared and uncollared bears were legally killed by permitted hunters each year (McLellan et al., 2017). By law, parts of all hunted bears had to be brought into an agency office for inspection where the deaths were recorded. Due to this Compulsory Inspection (CI) regulation, we assume all permitted hunter-killed collared and uncollared bears in the BC Flathead Valley were recorded in the government database. Bears that were killed by people for other reasons and reported were also recorded in the government database, but a number of human-caused mortalities are suspected to remain unreported. The difference in the ratio of bears killed by permitted hunters to bears killed by people for other reasons between the uncollared bears in the government database and the radiocollared sample provides an estimate of the number of unrecorded human-caused mortalities of uncollared bears. This method does not require a subjective estimate of whether or not the death would have been recorded by the agency based on the circumstances of each case, as done by McLellan et al. (1999). Nor does this method assume that the public will report deaths of radiocollared and uncollared bears with equal probability or that reporting rates are equal for all causes of mortality (Cherry et al., 2002).

### Study area

The North Fork of the Flathead River begins in BC, Canada, and flows southward where it crosses into Montana, USA, at N49° and W114.48° at an elevation of 1,165 m. The BC Flathead is 1,585 km^2^ and is BC Wildlife Management Unit (MU)4-01. The valley is about 12 km wide at the border and ranges of the Rocky Mountains rise to 3,000 m on the east and 2,200 m on the west. The valley in BC has been heavily logged. There has also been exploration for gas, coal, and minerals but no extraction. In 2016, there were no paved roads but 1,851 km of gravel/dirt roads in MU4-01 for an average density of 1.17 km/km^2^, although some of these were closed seasonally to the public or annually to hunters. Lower elevations were dominated by regenerating clearcuts and logging roads among some natural forests of lodgepole pine (*Pinus contorta*), western larch (*Larix occidentalis*), and Douglas fir (*Pseudotsuga menziesii*). Higher elevations were also dominated by regenerating cutblocks, but also Engelmann spruce (*Picea engelmannii*)-subalpine fir (*Abies lasiocarpa*) forests, slowly regenerating historical burns, avalanche chutes, rock, talus, and scree.

There were no permanent residents in the valley except three cabins within one km of the US border were occupied in the 1980s. The nearest highway and human settlement is about 40 km by gravel forestry road to the closest edge of MU 4-01 and >100 km to the far corners. The BC hunter survey suggests that an average of 2.6 hunter days per km^2^ were spent in pursuit of ungulates in MU 4-01 each autumn. Although some other MUs in BC where grizzly bears are hunted have almost four times the density of ungulate hunters, MU 4-01 has a higher ungulate hunter density than 83% of the MUs in BC where grizzly bears are hunted. Due to the distance from town, almost all hunting was based out of temporary camps with people staying mostly in wall tents, small trailers, slide-in campers, and a few cabins. The area has few summer or winter recreationists.

Both grizzly and black (*U. americanus*) bears were hunted in BC. There was a general open season in the spring and fall for black bears but grizzlies were only hunted in the spring. There was a draw for a limited number of grizzly bear permits for residents of BC with the average odds being one in 29.2 of getting a permit from 2007 to 2016. Non-residents of BC only hunted with a registered outfitter that had a quota, which can be less than one grizzly per year for a geographic territory with exclusive guiding rights.

## Methods

We examined the causes of mortality of grizzly bears using two datasets from MU 4-01. One was the monitoring histories and causes of death of radiocollared bears. The second was the BC government CI database of dead grizzly bears.

Radiocollared animals are not a truly random sample (Goodrich et al., 2008). To reduce possible bias, we captured bears at different times of year and used different methods. During both spring and fall we captured bears using both foot snares and culvert traps. We also darted grizzly bears from a helicopter in spring and summer. Capture and handling methods were approved by the BC Fish and Wildlife Branch. No grizzly bears were captured because of conflicts with people. We attached radiocollars with a canvas connector that decomposed allowing the collar to drop after a planned period that varied from <1 year to 6 years depending on the age and sex of the bear and thus ended a monitoring session for that individual. Most collars (217 of 255) were small brown or black very high frequency (VHF) collars and not easily seen. All collars contained a movement sensor which indicated the collar was dropped or the bear had died. We collared all female bears captured between 1979 and 2016 but we only put collars on a few males after 2003. Methods of tracking bears have been described in detail in McLellan (2015).

To estimate the implications of unknown fates, or when we lost contact with a radiocollared bear so it became a candidate for cryptic killing, we recorded the duration and fate of each monitoring session, as suggested by Treves et al. (2017). These fates were either: (1) dead bear; (2) rotted canvas so collar dropped off; (3) collar/battery failure but bear was detected later; (4) on-going monitoring, or (5) unknown fate. For each unknown fate, we recorded: (1) if the collar had stopped transmitting when the bear was hibernating or not, (2) the length of time the collar had been transmitting before it stopped and compared these to transmitting times before collar/battery failure of bears known to have survived monitoring sessions as well as theoretical battery life of the collar, and (3) in what jurisdiction or how far from MU 4-01 the bear was living when it was no longer located. Based on these factors, we determined the number of cryptic killing cases of collared bears that may have happened in MU 4-01.

Since 1976, all successful grizzly bear hunters in BC have been required by law to bring the skull and hide to an agency office where the location of kill and sex of the bear was recorded in the CI database. Grizzly bears killed for other reasons also had their sex, age, location of kill, and cause of death recorded in the same database. Bear deaths were categorized as permitted hunter kill, legal animal control, illegal kill, road kill, or rail kill.

We classified deaths of radiocollared grizzly bear the same as the BC database except natural mortalities were also recorded. All collared bears killed by permitted hunters were reported. For legal reasons, we reported the death or suspected deaths of all collared bears to Conservation Officers so these deaths were also in the CI database provided the carcass was found.

In this study, we compared the cause of death of our sample of bears when they were in MU 4-01 and wearing a functioning radio collar to the cause of death of uncollared bears in the CI dataset for MU 4-01, excluding all natural deaths. In particular, we compared the proportion of all human-caused mortalities of collared bears that were killed by permitted hunters to the same proportion for the uncollared bears in the CI database. Under the assumption that the radiocollared bears represented an unbiased sample of mortality and that all legal hunter kills were recorded in the CI database as is required by law, then the ratio of collared bears killed by permitted hunters to collared bears killed by people for all other reasons should be the same ratio for uncollared bears in the CI database. Deviations from this ratio would reflect the unreported human-caused mortality (Eqs. (1) and (2)).

(1) $${{{\rm{NHC}}} \over {{\rm{HC}}}}\, = \,{{{\rm{NHU}}ci\, + \,{\rm{NHUunreported}}} \over {{\rm{HU}}ci}}$$

That reduces to:

(2) $${\rm{NHUunreported}}\, = \,\left({{{{\rm{HU}}ci} \over\displaystyle {{{{\rm{HC}}} \over {{\rm{NHC}}}}}}} \right)\,-\,{\rm{NHU}}ci$$

where NHUunreported is the estimated number of non-hunting deaths of uncollared bears that were not reported, HU*ci* is the number of permitted hunting kills of uncollared bears in the CI database, HC is the permitted hunting deaths of collared bears, NHC is the number of non-hunting deaths of collared bears, and NHU*ci* is non-hunting deaths of uncollared bears in the CI database. Comparing the estimated number of unreported kills to the number of reported kills that were not by permitted hunters in the CI database provides the proportion of these kills that were unreported.

We used 10,000 randomizations with replacement of both the collared and uncollared sample simultaneously using the Microsoft Excel (Microsoft, Redmond, WA, USA) add-in program PopTools (Hood, 2011) to: (1) estimate the probability that the ratio of the kill by permitted hunters to bears killed by people for all other reasons was the same for the collared and uncollared sample, and (2) estimate 95% confidence limits around the number and proportion of the non-permitted kill of uncollared grizzly bears that were not reported.

## Results

In BC MU 4-01, 69 female grizzly bears were monitored for a total of 275 bear-years in 108 monitoring sessions (this includes recollaring of previously captured animals). Of these sessions 102 (94%) had known fates. Of the six unknown fates, four had collars outlast expected battery life, and one collar approaching the expected battery life failed while the bear was hibernating, leaving only one whose collar stopped transmitting with 1.8 years of theoretical battery life remaining so was a candidate for cryptic killing. Because 15 of 36 bears had collars stop transmitting in even less time but they were known to have survived the monitoring session, and this individual lived in the most remote portion of MU 4-01, we suspect her collar just failed prematurely, leaving no females with suspicious unknown fates.

In 81 monitoring sessions of 63 male grizzly bears covering 85 bear-years, 63 (78%) sessions ended with known fates. Of the 18 sessions with unknown fates, 11 ended with the bears dispersing to or otherwise living in either Montana or Alberta so were unlikely to have been cryptically killed in MU 4-01. Two collars stopped working when the bears were hibernating so they would not have been killed by people at that time. Four sessions ended with suspected cryptic killing; two in MU 01 and two in Montana (and thus not included in our analyses). In these cases, the radiocollars stopped functioning prematurely when the bears, which were known to have little fear of people, were located close to hunting camps in BC or residences in Montana. In one case in BC, a blood trail at the hunting camp was detected but neither the carcass or collar were found. The final unknown fate was of a 3-year-old male that lived in both BC and Montana. This bear could either have been cryptically killed, dispersed as young males often do (McLellan & Hovey, 2001), or the radiocollar could have failed prematurely; 9 of 36 collars on males that were known to have survived their monitoring session stopped working in less time.

Between 1979 and 2016, 14 female grizzly bears died in MU 4-01 while wearing a functioning radio collar (Table 1). Four were natural deaths, four were legally killed by grizzly bear hunters, three were killed in defense of life or property by ungulate hunters, one was maliciously shot and left on the roadside, and one was shot but we are unsure of the reason. Another female that died we suspect had been shot by an ungulate hunter. If this female was killed by a person as we suspect, then 4 of 10 (40%) human-caused deaths of collared females were by permitted grizzly bear hunters; 4 of 9 (44%) if this latter female died of natural causes.

**Table 1:** The number of radio collared and uncollared grizzly bears that were known to have died (suspected in parentheses) in the Flathead Valley of British Columbia, 1979–2016.

Sex	Cause of death	Collared	Uncollared in CI database
Male	Natural	1	0
	Legal hunting	6	45
	Nonhunting	4(2)	8
Females	Natural	4	0
	Legal hunting	4	26
	Nonhunting	5(1)	2
Both	Natural	5	0
	Legal hunting	10	71
	Nonhunting	9(3)	10

Between 1979 and 2016, 11 males died with functioning collars in MU 4-01 (Table 1). One was killed by another bear, six were legally killed by permitted hunters, one was shot at an ungulate hunters’ camp, one was killed in a neck snare set for wolves, and we are unsure why two were shot and left near the road side. In addition, were the two males suspected of being shot at ungulate hunters’ camps in MU 4-01. If these two males were cryptically killed as we suspect, then 6 of 12 (50%) human-caused deaths of collared males were by permitted grizzly bear hunters but this would be 6 of 10 (60%) if both of these males were not cryptically killed. When males and female data are pooled, then permitted hunters would have killed 10 of 22 (45%) of the collared bears when the three suspected cases are included but 10 of 19 (53%) if excluded.

The ratio between the number of permitted hunter kills to the number of human-caused mortalities but for other reasons is much different for the uncollared bears in the CI database than it was for the collared sample (*p* < 0.0001). In the CI database for MU 4-01, 26 of 28 (93%) of the uncollared females and 45 of 53 (85%) uncollared males were killed by permitted grizzly bear hunters or 88% when both sexes are pooled (Table 1). If uncollared bears were killed for the same reasons as the collared sample and the three suspected cases are excluded, then 84% (95% CI; 51–95%) of the deaths of uncollared bears by people without grizzly bear hunting permits would have been unreported. If three suspected deaths of radiocollared bears are included, then 88% (95% CI; 67–96%) of these human-caused deaths would have been unreported. These rates suggest that in addition to the 10 bears (95% CI, 5–16) in the CI database that were not killed by permitted hunters, another 54 (95% CI, 13–130) if suspected deaths of collared bears are excluded or 75 (95% CI, 26–197) if suspected deaths of collared bears are included, would have also been killed but not reported.

## Discussion

Based on the circumstances of death of each radiocollared individual, McLellan et al. (1999) estimated that 53% of the 57 known or suspected non-hunting human-caused mortalities of radiocollared bears would have been unknown to the management agency when data from 13 study areas were pooled. Of the four projects in BC, McLellan et al. (1999) estimated that eight of 13 (62%) non-hunting mortalities would have been unknown to the management agency if the bears had not been radiocollared. Some of these case by case subjective estimates of whether or not the death would have been reported were likely incorrect; uncollared bears killed for non-hunting reasons appear more unlikely to be reported than estimated by McLellan et al. (1999).

By comparing the ratio of bears killed by permitted hunters to those killed by people for other reasons between the radiocollared bears and the uncollared bears in the CI database, our estimates suggest that 84–88% of the bears killed by people for reasons other than permitted hunting were not reported in the Flathead study area. In another heavily logged and well accessed area in central BC, Ciarniello et al. (2009) had three collared bears killed legally by permitted hunters and five killed for non-hunting reasons while in the CI database, when legally killed conflict animals near human residences are excluded, 34 were legally shot by hunters and only five were killed by people without hunting permits. Although sample sizes were very small, these data also suggest that about 90% of bears killed by people for reasons other than permitted hunting were not reported.

The reporting rate for non-hunting human-caused mortalities would be slightly lower if the two bears in our study with unknown fates had been shot and their collars destroyed. We think such a fate was unlikely in these cases. We did, however, include two cases of suspected cryptic killing in our analysis. In these cases bears known to be habituated to human presence were last located near hunting camps and then their collars were never heard again.

Most non-hunting, human-caused grizzly bear mortalities were due to bears being attracted to hunting camps or during threatening encounters with ungulate hunters in the field. We suggest that there are features of the Flathead study area that makes reporting of non-hunter killed grizzly bears uncommon. First, the entire drainage is >40 km of gravel road from a Conservation Officer office and there is no cellular phone service so reporting a kill is time consuming and the chance of getting caught by a Conservation Officer for not reporting is presumably less than closer to a town. Second, there are no residences so little concern of a neighbor contacting an officer. In addition to the low reporting rate, the study area likely has a relatively high proportion of non-hunter kills because almost all ungulate hunters are based in field camps where grizzly bears are common as opposed to returning to their permanent residence after hunting.

Like most studies of grizzly bear mortality rates and causes (Knight & Eberhardt, 1985; McLellan, 1989, 2015; Mace et al., 2012; Garshelis, Gibeau & Herrero, 2005; Schwartz et al., 2006; Ciarniello et al., 2009) as well as for other species, we assume our collared sample reflects the uncollared population. Although a radiocollared sample may be the best available approximation (Goodrich et al., 2008), it may not be an unbiased sample (McLellan et al., 1999; Schmidt et al., 2015; Sönnichsen et al., 2017; Treves et al., 2017). Biases can arise from two sources; which animals are collared and if wearing a collar effects the probability that the animal will be killed. By catching bears using foot snares and culvert traps during spring and fall plus darting from a helicopter during spring and summer, we likely reduced any bias of sampling a disproportionate number of bold or careless individuals that may be more likely to be killed by people than more cautious bears. By limiting our analysis to MU 4-01, we also reduced the common tendency of collaring animals in less developed areas where densities (and survival) are higher than in more human-dominated landscapes (McLellan et al., 1999; Schmidt et al., 2015; Treves et al., 2017). Our focal area is relatively homogeneous regarding access and in particular, there are no settlements or paved roads. Although reducing bias, this restriction limits extrapolation. Reporting rates of grizzly bears killed for non-hunting causes may be different in unroaded wilderness or in areas with more human settlement and additional causes of mortality such as conflicts at residences and road or railway strikes.

Finally, we assume that wearing a collar had no effect on whether or not a person would kill a bear. If permitted bear hunters were more likely to refrain from shooting a bear because it wore a collar than were poachers, or ungulate hunters that either had a bear attracted to their camp or felt threatened by a bear, then our estimated reporting rate for non-hunter mortalities would be biased high. If poachers or ungulate hunters were more likely to refrain from shooting a bear because it wore a collar than were permitted bear hunters, then the reporting rate for non-hunting mortalities would have been even lower. For several reasons, this potential bias is likely less for grizzly bears in our study than for most other species. First, we almost exclusively used small, dark VHF collars that were relatively difficult to see against the dark, dense fur on a bear’s neck compared to studies using larger and more obvious GPS collars or on species with shorter neck hair. Second, grizzly bears usually live alone and therefore a hunter or poacher cannot simply select the uncollared individual or individuals in a group to shoot. Third, unlike most species, grizzly bears can be threatening and in such cases, a collar is unlikely to stop someone from shooting a bear in a hunting camp or in perceived self-defense. Finally, shooting a collared bear is legal and resident hunters had a one in 29 chance of drawing a permit and non-residents had paid an outfitter (plus other costs) so hunters were unlikely to refrain from shooting a bear that they were interested in just because it wore a collar.

Having such a high number of unreported, non-hunting human-caused mortalities in addition to the hunter kill is of concern for conservation. There is little doubt that the total human-caused mortality in this area has affected a variety of population processes. Fortunately, the procedure used in BC to calculate the number of bears that can be sustainably killed by all causes used a model to predict the unreported human-caused mortality for all population units (McLellan et al., 2017). For the Flathead, this model predicted a 2% annual unrecorded human-caused mortality rate which adequately compensated for this largely unknown factor. Additionally, the study area is productive and high densities of grizzly bears remain and are regulated by density-independent variability in food sources (huckleberry production) that affected reproduction more than by human-caused mortality (McLellan, 2015).

## Conclusions

In the Canadian portion of the Flathead Valley, about 88% (95% CI; 67–96%) of the uncollared grizzly bears that were killed by people for non-hunting causes were not recorded in the government mortality database. Managers and researchers should know that most bears killed by people for non-hunting reasons are unlikely recorded, at least in back-country areas. Reporting rates may be higher in areas nearer to human settlement. This lack of reporting will not only affect estimates of the total number of bears people kill but most other inferences regarding human-caused mortalities that rely on government databases. A low reporting rate of non-hunting human-caused mortalities may also be true for many species and this likelihood should be considered.

Our method to estimate the reporting rate of human-caused mortalities relies on an assumed 100% reporting of hunter killed bears. Where grizzly bears are not hunted, other forms of human-caused mortality with 100% reporting, such as bears killed by conservation officers in conflict situations, could be used as the known sample instead of hunter-killed bears to estimate reporting rates of other forms of human-caused mortality.

## Supplemental Information

10.7717/peerj.5781/supp-1 Supplemental Information 1 Collared and uncollared bears killed by people in the Flathead Valley, BC, 1979 to 2016.

## Additional Information and Declarations

### Data Availability

The following information was supplied regarding data availability: The raw data are provided in a Supplemental File.
